# Strategic Training Executive Program 2.0: A Leadership and Change Management Program for Health Supply Chains in Low- and Middle-Income Countries

**DOI:** 10.9745/GHSP-D-23-00365

**Published:** 2025-05-09

**Authors:** Patricia Bobo, George Bray, Kevin Etter, Namrata Singh

**Affiliations:** a Ministry of Health, Lusaka, Zambia.; b International Federation of Pharmaceutical Wholesalers Foundation, Paducah, KY, USA.; c Consultant, Gavi, the Vaccine Alliance, Elizabethtown, KY, USA.; d Empower School of Health, Haryana, India.

## Abstract

People that Deliver’s vision is “a world where health supply chain workforces are empowered and equipped to optimize health outcomes by improving access to health commodities.” However, health supply chain management (HSCM) is not a recognized profession in many low- and middle-income countries (LMICs). HSCM professionalization efforts are essential if health outcomes are to be significantly improved in these countries; this means transforming supply chain management into a recognized profession of the highest integrity.

The Strategic Training Executive Program (STEP) was created in 2016 to address prevailing leadership style gaps. Since then, STEP has undergone rigorous revisions to improve its applicability, scalability, and usefulness in LMICs. To date, STEP—in all its forms—has been delivered to 30 countries and contributed to building the supply chain capacities of more than 600 health supply chain professionals.

This case study discusses the evolution journey of designing and sustaining the second generation of STEP (STEP 2.0). This journey is an innovative illustration of how diverse yet motivated organizations collaborated during a global lockdown and health emergency to reimagine a program recognized by all as essential for post-pandemic supply chain systems.

## Abstract (precis)

STEP 2.0 is an innovative approach to developing leadership and change management competencies that will enable local supply chain management professionals to contribute to commodity and medicine availability, leading to improved health outcomes in low- and middle-income countries.

## Key Messages

- Optimizing leadership competencies of supply chain management (SCM) leaders in low- and middle-income countries (LMICs) ultimately contributes to enhanced commodity and medicine availability with improved health outcomes.
- The Strategic Training Executive Program (STEP) was developed as a capacity-building program to address supply chain operations challenges in LMICs, including lack of formal training of the supply chain management (SCM) workforce and inadequate leadership approaches, and help strengthen the SCM workforce by increasing SCM competencies.
- The program was revised to STEP 2.0, which emphasized a collaborative problem-solving methodology, engaged private-sector coaches, and expanded partnerships with other donors and implementing partners.
- Step 2.0 has been successfully implemented in Ethiopia to apply collaborative problem-solving and team leadership concepts in last-mile delivery interventions and territory expansion, in Zambia to help upgrade a logistics management information system to enable health districts to extend their initial system training to health centers within their districts, and in Rwanda to facilitate the use of multisectorial, cross-functional collaborative teams at each phase of the innovative medical drone program.

## BACKGROUND

People that Deliver (PtD) is an initiative that was conceived in 2011 when 79 institutions came together at the World Health Organization headquarters and pledged to strengthen the capacity of the health supply chain workforce while promoting the professionalization of supply chain roles within the health systems. PtD is hosted by and housed within the UNICEF Supply Division.

In 2011, PtD published a literature review that identified critical gaps in management skills and competencies throughout public health supply chain systems in low- and middle-income countries (LMICs).^1^ One key indicator seemed to explain why these gaps existed—the severe lack of trained and skilled professionals to manage health supply chain commodities was, in part, restricting access to health care commodities.^1^ When health workers are not adequately trained to manage health commodities, wastage and inefficiencies result in poor service delivery and hamper access to critical medicines and supplies, especially at the community level and in vulnerable settings. Effective health care leadership, particularly as it pertains to commodity and supply chain management (SCM), is central to strengthening health services and systems that maintain good-quality and equitable health care service delivery.

One of PtD’s strategic goals is the global acknowledgment of the critical role of robust supply chains in ensuring positive health outcomes, particularly as it pertains to ensuring the availability of quality medicines, vaccines, tests, and laboratory equipment for health delivery and system strengthening, especially at primary health care levels. To reach this goal, it is important to map all critical supply chain tasks and the requisite knowledge, skills, and abilities required to achieve these tasks, with the end goal being to ensure professional education and certification to develop these competencies and build qualified staff cadres.^2^

In 2013, PtD began in earnest to research and document the requisite skills and attributes for a professional supply chain manager supporting public health systems. An initial framework was developed that defined the skills and attributes, which were then organized into 6 process domains: 4 technical domains (selection and quantification, procurement, storage and distribution, and use) and 2 management domains (resource management and professional and personal) were established. The initial framework was reviewed by 120 participants at a PtD leadership workshop in Copenhagen in October 2014. It was then presented to an International Association of Public Health Logisticians (IAPHL) forum to a larger group of reviewers for a scoring and prioritization exercise. Finally, in February 2015, the *People that Deliver Health Supply Chain Competency Framework for Managers and Leaders* was published.^3^

A collaboration between PtD and IAPHL on challenges related to human resources for SCM (HR4SCM) led to the publication of an analysis incorporating insights from 103 contributors representing 24 countries and highlighting 3 main areas of concern (Box 1) related to HR4SCM and the professionalization of SCM.^4^

BOX 1 Gaps in Human Resources for Supply Chain Management and the Professionalization of Supply Chain Management

- Human resources as a barrier to health supply chains
  - Lack of supply chain strategy
  - Limited focus on components of supply chain management (SCM)
  - Unclear lines of responsibility
  - Insufficient education
  - Lack of supply chain resources
  - Limited focus on human resources
- Taking a systemic approach to human resources for supply chain management
  - Need for SCM leaders and strategy
  - Call for professionalization
  - Define and support the SCM workforce
  - Multiple entities engaged in SCM
  - Weak advocacy and policy
  - Aspects of human resources for supply chain management are siloed
- Pre-service education and continual professional development as a critical component [of professionalization of SCM]
  - Needs-based approach required
  - Inadequate current approaches
  - Relevance of generalized SCM education
  - Insufficient in-service training
  - Inadequate political understanding

Source: People that Deliver 2014.^4^

Around the same time, Gavi, The Vaccine Alliance began working on a supply chain strategy that comprised 5 strategic initiatives (people and practice, data for management, cold chain equipment optimization, system design, and private-sector engagement). The paper highlighted the lack of formal training among the SCM workforce and the lack of structure and systems in place to share good practices. It was aimed at exploring personnel profiles in both the public and private sectors to gain a comprehensive understanding of the expertise, authority, and resources required for effective SCM to address HR challenges in immunization supply chains.^5^^,^^6^ This paved the way for an innovative public-private partnership to strengthen HR4SCM.

A collaborative working group of the People and Practice and Private-Sector Engagement initiatives called the People and Practice Priority Working Group (with membership from the U.S. Agency for International Development, [USAID], PtD, Gavi, and UNICEF) was established and began an initiative called “HR in SCM.” It was immediately recognized that compounding the lack of supply chain professionalization was the prevalent leadership style in LMICs: a traditional and outdated “command and control” methodology, where the leader retained all knowledge, authority, and decision-making power.

Compounding the lack of supply chain professionalization was the prevalent command control leadership style in LMICs, where leaders retained all knowledge, authority, and decision-making power.

Today, the lack of trained, skilled, and motivated professionals in the supply chain workforce persists,^7^ and the assumption is that the command control style of management is inadequate when confronted with the complexities of current health supply chain system challenges.^8^ Rather, private-sector best practices reveal that team-based collaborative leadership provides nimbleness and speed to problem-solving.^9^

Building on these findings, the working group established a strategy to lead the development of a capacity-building program that drew on best practices from the private sector and incorporated contemporary 21st-century leadership techniques, focusing on teamwork and collaborative problem-solving. Gavi leveraged the guidance of a secondee from United Parcel Service, Inc. (UPS) and the professional competencies defined in PtD’s framework to build a unique leadership development program called the Strategic Training Executive Program (STEP), which emerged as a strategic response to address these supply chain operations challenges and as a solution to strengthening the SCM workforce.

During the development of STEP, 3 important factors were considered: (1) the level of national management targeted by the program; (2) the nuances of working with sovereign government ministries; and (3) the use of specific words and their varying interpretations (for example, the word “leader” became problematic early on).

This program case study attempts to highlight the value of unique capacity-building programs, like STEP, which leverage private-sector engagement in ensuring professionalization and strengthening of the supply chain workforce in LMICs.

## EVOLUTION OF THE STRATEGIC TRAINING EXECUTIVE PROGRAM

### The First Generation

In concept and in design, STEP is a leadership development program designed to “upskill” key decision-makers in supply chains in LMICs by enhancing their strategic problem-solving skills.

The first generation of STEP adapted the best (or most promising) practices of highly successful private-sector businesses into a set of learning modules by Skillsoft. The STEP development team worked with Skillsoft to develop an experiential program centered on adult learning principles that had been widely adopted in the private sector. The program’s underlying thesis is rooted in the basis of experiential learning, which is learning by doing, and states that “adults, particularly those well into their careers, learn best through experience and from each other.” This approach aligns with Kolb’s experiential learning theory, which states that adults learn in 4 phases: concrete experience, reflective observation, abstract conceptualization, and active experimentation.^6^ The STEP methodology imbibes these phases through reflection, exposure to technical concepts, and active application of leadership and change management skills in a workplace setting. The program is shaped around collaboration and team-building to develop core competencies in 5 areas: (1) building a team, (2) planning for success, (3) successful execution through teams, (4) sustaining relationships, and (5) evaluation of success.

STEP is shaped around collaboration and team-building to develop core SCM competencies.

The first generation of STEP was delivered in 3 program phases:

- Phase 1: Distance learning modules, where program delegates complete a series of 4 online modules independently. Duration: 1 month.
- Phase 2: 5-day in-person workshop, where delegates worked together in 3–5 member teams to learn critical leadership and team-building skills through a multifaceted workshop.
- Phase 3: Capstone project, where each team of delegates is paired with a private-sector mentor to complete a work-based project in their area of responsibility or influence by applying the skills gained in the preceding 2 phases. Duration: 3 months.

**FIGURE 1:**
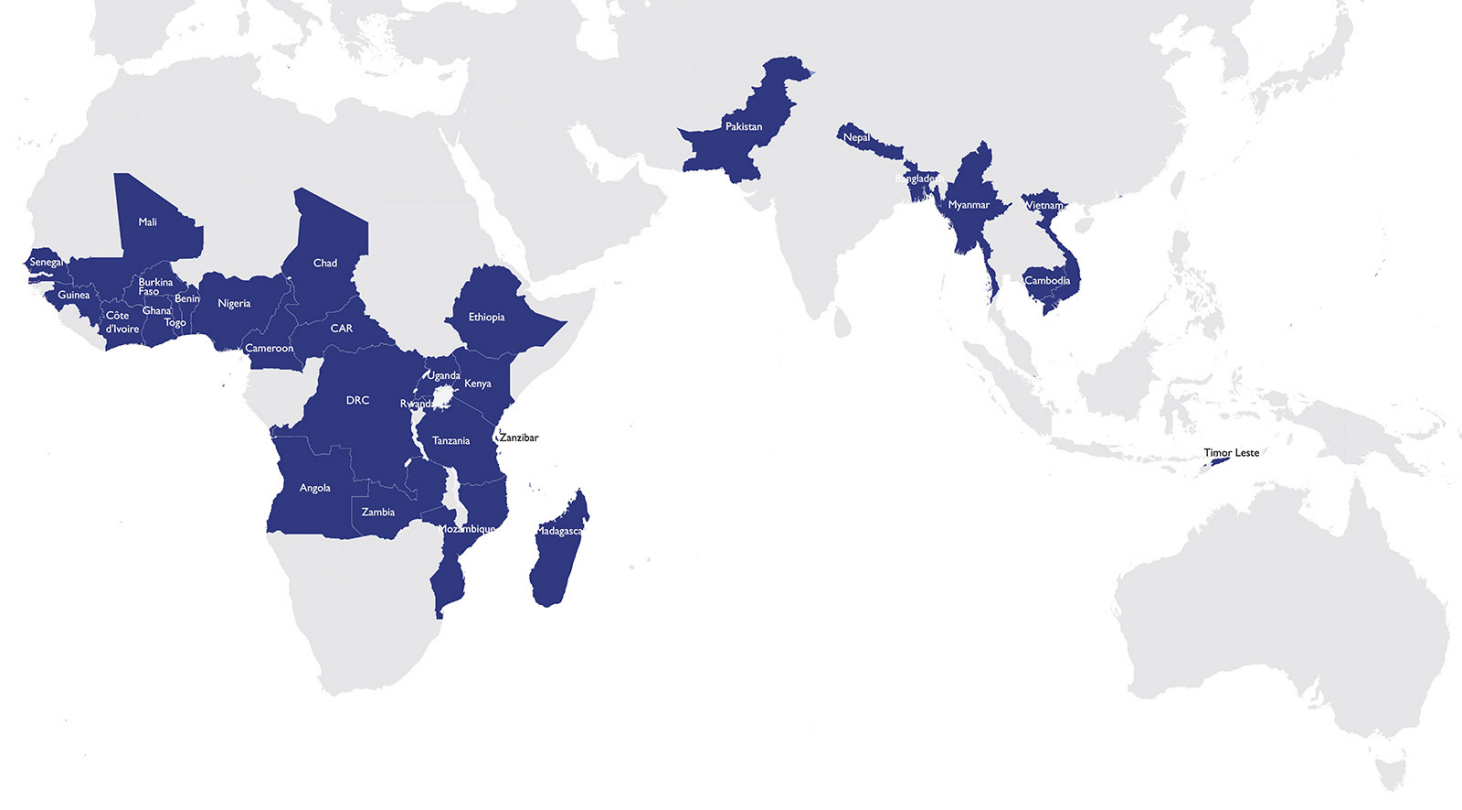
STEP Participants’ Countries of Origin From Inception to 2024 Abbreviations: CAR, Central African Republic; DRC, Democratic Republic of the Congo; STEP, Strategic Training Executive Program.

In December 2015, STEP was first presented in Kigali, Rwanda, to a group of East Africa Community supply chain leaders with representation from Rwanda, Uganda, Kenya, and Tanzania, and was well received. In the years that followed, 11 additional STEP training courses were provided by Gavi. Cumulatively, the first generation of STEP was offered 12 times to 341 delegates from 21 African nations and Pakistan. Figure 1 shows the countries where STEP (2016–2020) and STEP 2.0 (2021–present) were offered.

In 2019, Gavi commissioned a midterm review (MTR) of the program. Following validated protocols, the research team conducted a desk review using a document review grid and Internet search tools (Google, PubMed, and Scholar). They also deployed online surveys, conducted in-depth stakeholder interviews, and performed mapping and analysis of social networks. The team, led by an independent consultant from GaneshAid, determined that the program was successful but needed to be refreshed and updated (unpublished) and offered several recommendations (Box 2).^10^

BOX 2 Recommendations From the Midterm Review of the Strategic Training Executive Program

1. Reinforce Strategic Training Executive Program (STEP) strategic planning and program management.
  - The courses reviewed by the midterm review were not driven by a specific Gavi strategy but were offered opportunistically on request.
  - Gavi relied on private-sector secondees to manage the program.
2. Optimize STEP’s partnership effectiveness.
  - It was recognized that the program offered opportunities to deepen partnerships with the private sector, other donor organizations, and implementing partners.
  - The report recommended more emphasis be placed on the partnership aspects of the program.
3. Address perception of conflict of interest.
  - Because the program relies heavily on private-sector engagement, the adoption of robust conflicts of interest policies was recommended.
4. Redesign and update STEP instructional materials.
5. Build long-term partnerships with implementing partners.
  - The report reflected the difficulty Gavi had had in both managing and implementing the program.
  - The lack of a set of certified implementing partners hindered the sustainability and scalability of the program.
6. Develop and nurture a STEP community of practice that comprised alumni.
7. Diversify sources of funding beyond private-sector support to ensure sustainability and scalability.

Gavi decided to extend the program until 2024 and to address the recommendations from the MTR. In 2019, Gavi engaged an independent consultant to lead the reengineering of the STEP program.

### Development and Application of the Second Generation

To address the MTR recommendations, PtD convened a 2-day workshop in Geneva, bringing together representatives from PtD, Gavi, the Global Fund, UNICEF, USAID, and UPS. The workshop focused on 2 prevailing leadership development programs by PtD’s coalition partners—Gavi’s STEP and USAID’s Transformational Leadership program. It was recognized that the programs were similar and targeted the same cohort—Ministry of Health employees who were responsible for health supply chain leadership and management at all levels of the health systems.

The workshop participants concluded that the programs were similar because they were both built around the leadership and personal development competencies detailed in the PtD Health Supply Chain Competency Framework.^3^ It was collectively agreed that for the sake of efficiency and to reduce confusion, only 1 program should be offered.

At the time, as updates were being planned for both programs, an opportunity emerged to merge the 2 programs by consolidating material from both. Given its 13 implementations and growing reputation, STEP was recognized as a more established program, and a decision was made to retain the STEP name. Subsequently, the name STEP 2.0 was adopted to signify a new version of the program, which would differ significantly from its predecessor.

To diversify funding sources, the 3 donor organizations (Gavi, The Global Fund, and USAID) signed a collaboration framework that enabled joint sponsorship and support of STEP 2.0. IFPW would later sign this framework, which outlined the governance policies for the program and established the PtD STEP 2.0 hub to oversee its governance. This multidonor approach is a fundamental cornerstone to the long-term sustainability of the program.

The content development team was expanded to include more international nongovernmental organizations, private-sector partners, academia, and STEP alumni representatives from Kenya, Uganda, and Benin. The group systematically revised the STEP 2.0 training material, incorporating material from reputed and established leadership programs as well as alumni insights to ensure that the material addressed contextual leadership challenges in LMICs. One key change in the content strategy for STEP 2.0 was the inclusion of a proven problem-solving methodology to develop leadership competencies. This shift repositioned STEP 2.0 from being a training resource to a dynamic tool for impact and change.

One key change in the STEP 2.0 content strategy was including a proven problem-solving methodology to develop leadership competencies.

### Virtual Strategic Training Executive Program 2.0

The onset of the COVID-19 pandemic, which resulted in travel restrictions and postponed physical meetings, coincided with the initiation of content revisions of STEP 2.0 material. Consequently, the content development team was compelled to engage in a virtual development and collaboration process. At the end of 2020, the program had undergone a comprehensive expansion and was ready for implementation. However, even though travel restrictions persisted, there was a strong demand for STEP 2.0. Consequently, Gavi financed the adaptation of STEP 2.0 to a virtual format. This version, called vSTEP 2.0, aligned with the norms of distance and home-based work practices that were adopted during the pandemic.

vSTEP 2.0 retains the first program preparation phase and combines the workshop and transformation challenge phases. Instead of conducting a day-long virtual workshop across 5 days, vSTEP 2.0 comprises 13 2-hour virtual sessions delivered over a 20-week timeframe. This restructuring has proven to be effective, especially when in-person contact is restricted.^11^ The virtual program has the advantage of being cost effective and is able to support larger cohorts of participants.

In early 2022, Gavi sponsored vSTEP 2.0 in Zambia. The Zambia Ministry of Health, working closely with Yale’s Global Health Leadership Initiative, selected 30 participants responsible for the performance of the vaccine supply chain network across Zambia, promoting alignment with the government’s strategic priorities for health systems strengthening and accountability for engagement. The participants were selected from the district, provincial, and national levels of the health system and across all 10 provinces in Zambia.^10^ Participants selected transformation challenges, such as improving COVID-19 vaccination coverage, use of immunization data, vaccine stock-outs and wastage, management of cold chain equipment, and improving government funding and staff retention.

vSTEP 2.0 in Zambia revealed promising results in participant engagement even with a virtual deployment. Through qualitative interviews to gather participant feedback, the Global Health Leadership Initiative found that participants applied vSTEP2.0’s leadership concepts to improve the COVID-19 response in Zambia by equipping participants with critical analytic and problem-solving skills to pinpoint root causes of challenges within the cold chain and COVID-19 vaccine distribution chains and practical strategies that they applied to address supply issues and space constraints that were prevalent during the pandemic.

Through biweekly coaching sessions, coaches helped participants identify the focal root cause and develop the implementation and evaluation plans of their transformation projects. Participants also reported that the application of Kotter’s methodology, which they learned from vSTEP 2.0, was instrumental to adopting a structured approach to the complex supply chain problems faced during the pandemic.

vSTEP 2.0 was directly attributed to improving their understanding of supply chain challenges, equipping them with structured leadership skills, and providing an avenue to apply these skills to improve supply chain performance and emergency response in Zambia.

### Comparison of the Different Versions

The evolution of STEP from its first generation to the second generation arose from the need to improve the program, address the MTR recommendations, and provide a more robust capacity-building program to the target audience. vSTEP 2.0 virtualized the components of STEP 2.0 and is considered an extension of STEP 2.0. Although changes were made, the following core elements of STEP were retained across all versions.

- The program’s focus is on team-building and leadership with an overall goal to increase SCM competencies.
- The objective of the in-person workshop (virtual sessions in vSTEP 2.0) is to learn and practice team leadership and collaboration.
- The workshop is facilitated by expert facilitators and supported by private-sector coaches.
- The workshop provides experiential sessions via plenary, stakeholder, and individual presentations, as well as case studies, facilitated discussion, team exercises, and breakouts.

Major differences between the first generation of STEP and STEP 2.0/vSTEP 2.0 include the following.

- Transition from a leadership skills development training course emphasizing team-building and leadership to a collaborative problem-solving tool applied through team-building and leadership.
- Expansion of the donor base from solely Gavi to a multidonor platform.
- STEP management shift from private-sector secondees through Gavi to implementing partners determined by donors.
- Translation of the content of STEP 2.0 into French, Portuguese, and Spanish to reach a broader audience.
- Revision of nomenclature from “delegates” to “participants” to signify a transition from mere representation of their country or areas of responsibility to active involvement in leading change within their respective regions. Another change was from private-sector “mentorship” to “coaching.”
- Change in Phase 1 from self-directed learning focused on skill development to self-directed preparation for the success of the entire program.
- In Phase 3, the transition from showcasing lessons learned during training via a capstone project to demonstrating leadership and change management competencies by successful resolution of workplace issues/problems, referred to as “your transformation challenge.” This was implemented with the inclusion of action plans drafted and implemented by participants with guidance from private-sector coaches.

### Private-Sector Volunteer Coaches: The Key to Success

One of the unique features of STEP and STEP 2.0 is the engagement and partnerships with private-sector health supply chain professionals. These professionals serve as “skilled volunteers” from the private sector and act as “coaches” to participants. One coach is typically responsible for providing guidance, experience, and assistance to a team of 4–6 participants. Coaches are selected and sponsored by their employers, who receive guidelines on the characteristics of successful STEP 2.0 private-sector coaches. Once selected, the coaches are provided with program materials and undergo an onboarding/orientation training session.

One of the unique features of this program is the engagement and partnerships with private-sector health supply chain professionals.

The rich interaction between international, regional, and local private-sector coaches provides a unique and enriching experience for participants, simultaneously cultivating North-South and South-South cross-learning. Each type of coach offers distinct advantages. Local or regional coaches have a more comprehensive understanding of contextual challenges, whereas international coaches bring a global perspective to be considered within the local context.

STEP employs distinct incentives to ensure continued and sustained private-sector engagement, which include employee engagement; executive development; succession planning; and contribution to furthering environmental, social, and governance and corporate social responsibility goals.

These objectives are pivotal for large organizations and can be achieved by supporting STEP leadership programs through the provision of facilitation and coaching expertise. International coaches, in particular, derive an additional benefit, as their exposure to other parts of the world fosters an appreciation and understanding of the challenges faced by LMICs.

Formal and informal surveys and interviews capturing participants’ perceptions of the coach’s impact consistently reflect strong support for this aspect, often highlighting it as the single differentiating feature of the program. The value of private-sector coaches is further highlighted by anecdotal testimonials from participants of previous STEP cohorts. A pharmacist from Uganda highlighted that access to a network of coaches and peers from several regions was critical to cross-learning and knowledge-sharing. Under the guidance of facilitators and coaches, professionals from Kenya understood the importance of team dynamics, emotional intelligence, and adaptation of leadership styles for differing situations.

Public-sector professionals were also exposed to best practices from the private sector, including the use of direct deliveries to address logistical challenges, collaborative tools when working with diverse teams, fostering a sense of ownership among teams, and unique problem-solving skills. Participant testimonies describe the impact of STEP on supply chain functions in terms of improvement in stock control, use of an electronic logistics management information system to address poor data quality challenges, micro-planning and performance monitoring in immunization supply chains, improved access to service delivery points, and reduction in service disruptions. Through hands-on private-sector engagement, skill and knowledge transfer is facilitated, contributing to the upskilling of the public health workforce. This is further corroborated by post-program surveys and in-depth interviews.

## AN EXAMPLE OF THE STRATEGIC TRAINING EXECUTIVE PROGRAM 2.0 IN ACTION

In 2023, 25 leaders and managers from Rwanda Medical Supply Ltd (RMS)—the public organization charged with ensuring the country’s access to health commodities—participated in and graduated from STEP 2.0. Every participant was from RMS, offering a real opportunity to change organizational culture. When a significant number of staff understand the STEP 2.0 principles and approach and participate together in the program, staff develop a mutual understanding of the best way to overcome challenges and lead their teams.^11^ The RMS leadership used the program preparation phase to define the organization’s main challenges. The STEP 2.0 facilitation team and coaches then worked with them to identify the top 6 supply chain transformation priority areas to focus on throughout the program.

The workshop phase of the STEP 2.0 program provides an opportunity for the participants to work together with the coaches to improve their leadership and problem-solving skills and work through the change management process. On this occasion, the private-sector coaches were provided by AmerisourceBergen (now Cencora), GlaxoSmithKline, Johnson & Johnson, Merck, and Pfizer.

In Rwanda, the participants worked together to find solutions to organizational challenges, including data integrity, personnel retention, private-sector engagement, and warehouse management. Bringing together colleagues from various teams with different roles to tackle real problems—with the support of private-sector expert coaches—offered a unique opportunity for RMS leaders to enhance their leadership abilities and take steps to address organizational challenges.

In Rwanda, the program participants worked together to find solutions to organizational challenges, including data integrity, personnel retention, private-sector engagement, and warehouse management.

All participants who attended the workshop said they would highly recommend the STEP 2.0 program to their procurement and SCM colleagues.

A number of predefined indicators were tracked by the implementing partner (Empower School of Health) to contribute to the monitoring and evaluation of the STEP 2.0 program. These indicators included participant details, participation, graduation rate, leadership competencies before and after the program, participant ratings of the program, and suggested improvements. Competency centered on 5 categories: lead, shape, plan, act, and evaluate. The difference between the first self-evaluation (program preparation assignment 4, completed on July 27, 2023) and the last self-evaluation (post-your transformation challenge survey form, last response received on December 14, 2023) was measured. There was an overall 8% weighted average increase across all competency ratings in this 6-month period (unpublished data).

During the workshop, 16 participants were asked to select 3 competencies to target for development during the your transformation challenge phase with the support of their coach. Of these, 14 completed the self-evaluation at all reporting periods (baseline, midline, and endline). There was a 21%, 19%, and 23% weighted average increase in competency rating from midline to endline for the first, second, and third competencies targeted for development, respectively. Because 21% (the average of the 3) is much higher than the overall average of 8% seen earlier, it was concluded that when a competency is prioritized and targeted for development, an increase in competency rating is more likely to be achieved.

## DISCUSSION

**FIGURE 2:**
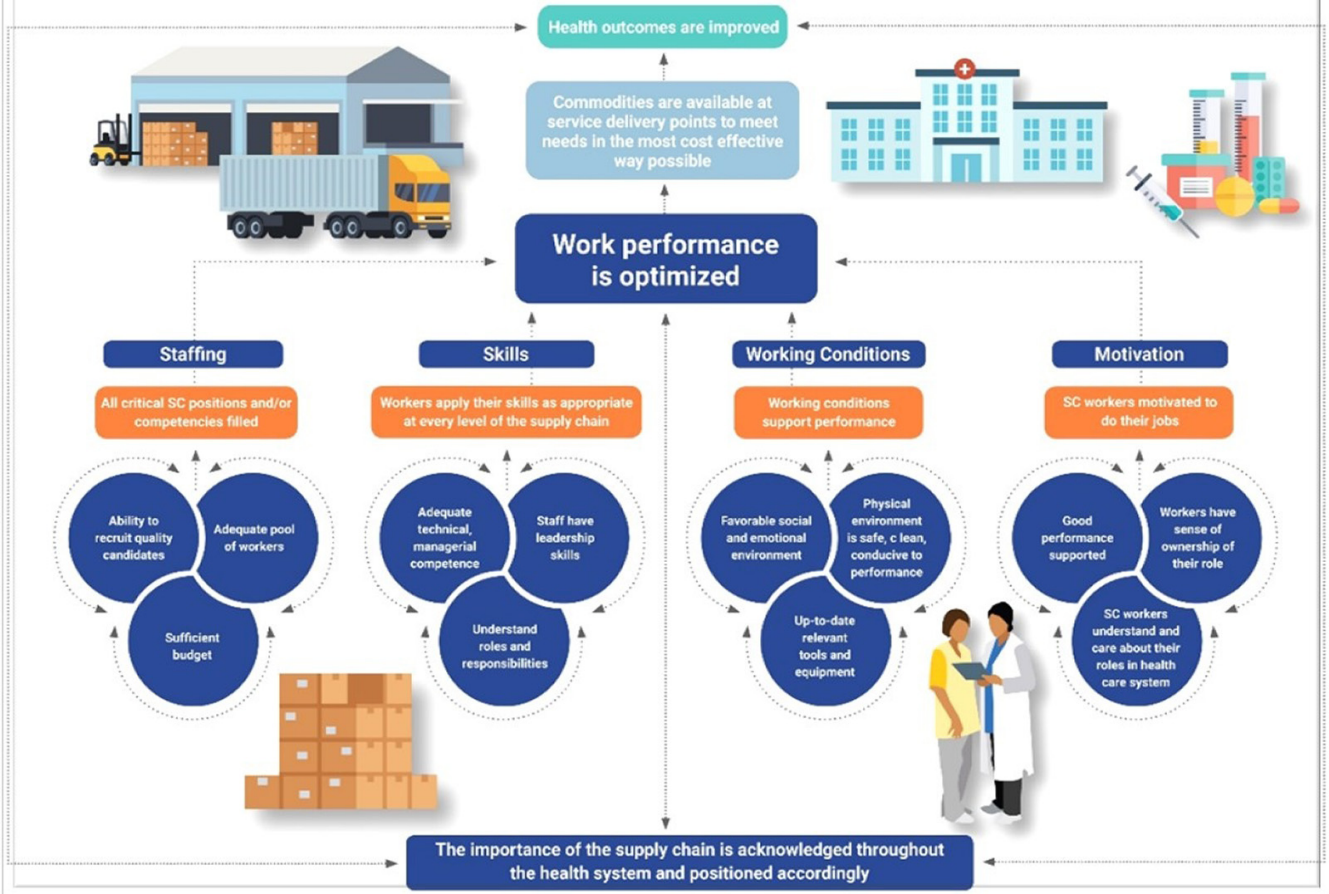
The Four Pathways of People that Deliver’s Human Resources for Supply Chain Management Theory of Change^12^ Abbreviation: SC, supply chain.

PtD and the STEP 2.0 donor organizations regard the program as a component of a broader set of interventions that LMICs can use to develop a holistic approach to HR4SCM and supply chain systems strengthening interventions. The guiding principles of these interventions are laid out in PtD’s Theory of Change^12^ (Figure 2), which details the pathways and tasks required to fully establish a professionalized SCM workforce.

STEP 2.0 strengthens the leadership competencies and problem-solving capacity of supply chain workforces with examples of its implementation in LMICs, including Ethiopia, Zambia, and Rwanda. In Ethiopia, STEP 2.0 collaborative problem-solving and team leadership concepts were applied in last-mile delivery interventions and territory expansion. In Zambia, STEP 2.0 was leveraged in restarting a stalled logistics management information system upgrade, enabling health districts to extend their initial system training to health centers within their districts. In Rwanda, STEP 2.0 facilitated the use of multisectorial, cross-functional collaborative teams at each phase of the innovative medical drone program. This is further supported by program alumni testimonials that underscore the positive impact of STEP 2.0 on forecasting and stock management, mentoring, and improving confidence and leadership skills. STEP 2.0 catalyzes SCM professionalization by encouraging the adoption of inclusive, collaborative leadership methods and embodying continuous improvement, with the end goal of improving overall health outcomes in LMICs.

The program’s commitment to continuous improvement extends inwardly, with PtD acting as the STEP 2.0 hub to identify and address program challenges with private-sector volunteer recruitment, support accreditation of implementing partners, develop measurement and evaluation tools, and ensure cohesive funding mechanisms.

Depending on the country/region of implementation, the recruitment of private-sector skilled volunteers can be challenging. Volunteer coaches are sourced from local or international private-sector organizations. In considering local representation, the deciding factor is whether the country has a viable private-sector supply chain network of providers—many countries do, but some do not. From an international volunteer perspective, the challenges relate to travel costs and the political stability of the country. To address these challenges, the hub has taken several steps to develop, implement, and sustain a roster of private-sector organizations willing to provide skilled volunteers from their employees.

The complexity of the program requires an implementing partner that is familiar with the program; thus, certification and accreditation of the partner are required. The partner certification process is currently the shared responsibility of the donor organizations and PtD. To address this challenge, PtD has recently commissioned the development of an institutionalization and accreditation strategy to analyze the feasibility of endowing regional institutions with the responsibility of overseeing the program.

A lack of measurement and evaluation tools for the first generation of STEP impeded donors’ ability to document the impact of the program. Moreover, measuring the direct impact of leadership and management skills strengthening is challenging without predetermined and baseline operational metrics and due to the complex nature of measuring the impact of complex development programs like STEP. To address these challenges, PtD developed a measurement and evaluation framework in 2023, with corresponding application tools and reports, to better demonstrate the impact of the program with each implementation. STEP 2.0 has since increased its focus on operational impact measures, ensuring that baseline metrics are established and tracked and exploring practical and participative tools for outcome-based learning, such as outcome harvesting, to identify, monitor, and learn from the changes STEP 2.0 influenced.

To date, the majority of the STEP programs offered have been donor driven rather than country driven. The hub serves to coordinate resources and activities across the multidonor platform, ensuring efficiency and judicious use of resources, as well as generating country-driven demand.

In summary, STEP 2.0 is a dynamic program that reinvents and adapts itself to differing contexts and challenges, imbibing the concepts it espouses and emphasizing the need for transformative and adaptive approaches in existing supply chain systems. Through the implementation of STEP 2.0, leadership and change management competencies developed during STEP, as well as key learnings from the private sector, become intrinsic and can be applied to other HSCM issues. Thus, the program contributes to professionalizing SCM workforces, catalyzing institutional capacity-building, and ensuring resilient supply chain systems.

The STEP methodology leverages international facilitators and private-sector coaches, who are instrumental to achieving the pivotal objective of optimizing HSCM leadership competencies. Survey results, qualitative interview findings, and testimonials from past STEP cohorts underscore the value of private-sector coaches, showcasing tangible improvements in supply chain functions due to learnings from the private sector. Participants’ cross-learning experiences, awareness of best practices, and application of innovative solutions in their respective countries highlight the program’s transformative impact. Initiatives like STEP not only enhance individual competencies but also contribute significantly to the broader goal of ensuring the availability of commodities and medicines, ultimately leading to improved health outcomes in LMICs. However, the ultimate success of interventions like STEP 2.0 relies on countries prioritizing supply chain professionalization in their strategic plans and operational budgets.

Participants’ cross-learning experiences, awareness of best practices, and application of innovative solutions in their respective countries highlight the program’s transformative impact.

## CONCLUSION

Twentieth-century leadership methods are ill suited to address complex 21st century HSCM; a new approach is needed. STEP 2.0 is at the forefront of a concerted effort to draw attention to the required leadership and management competencies for HSCM: this is an approach that PtD, along with its STEP 2.0 collaborators, believes to be pivotal to professionalizing the supply chain workforce. The program is the fruit of a collaboration between donor organizations, the private sector, and development partners. It is an example of diverse actors pooling their resources and expertise to develop a solution to overcome the obstacle of ineffective leadership in the health supply chains of LMICs.

Optimizing HSCM leadership competencies will contribute to commodity and medicine availability and, in doing so, will contribute to improved health outcomes in LMICs and the achievement of Sustainable Development Goals and universal health coverage.

PtD advocates for intensifying the current momentum STEP 2.0 has gathered thus far. This entails securing additional funding for STEP 2.0 programs to enable and equip even more supply chain leaders to lead their teams through supply chain challenges. It also calls for more countries to express an interest in STEP 2.0 and make donors aware of this interest. PtD also calls for greater engagement from the private sector, as the program relies on companies to provide volunteer coaches who generously share their expertise. By deepening this collaborative effort, more countries can leverage STEP 2.0 and use the skills they develop throughout the program to strengthen their supply chains and improve access to health commodities, thereby enhancing public health outcomes.

## Notes

**Peer Reviewed**

**First Published Online:** September 12, 2024.
